# Characteristics of Composite Materials of the Type: TPU/PP/BaTiO_3_ Powder for 3D Printing Applications

**DOI:** 10.3390/polym15010073

**Published:** 2022-12-24

**Authors:** Romeo Cristian Ciobanu, Cristina Schreiner, Mihaela Aradoaei, Gabriela Elen Hitruc, Bogdan-George Rusu, Magdalena Aflori

**Affiliations:** 1 Electrical Engineering Faculty, “Gheorghe Asachi” Technical University of Iasi, Dimitrie Mangeron Bd., 67, 700050 Iasi, Romania; 2 Petru Poni Institute of Macromolecular Chemistry, Aleea Gr. Ghica Voda, 41A, 700487 Iasi, Romania

**Keywords:** composite materials, thermoplastic polyurethanes, barium titanate, the thermogravimetric analysis

## Abstract

Composite materials are materials with anisotropic properties that are created by combining several different components in a way that allows the best qualities of each component to be used. In this paper, raw materials were used to obtain composite materials of the type TPU/PP/BaTiO_3_ powder. The thermogravimetric analysis, dynamic differential calorimetry, and scanning electron microscopy were carried out. The preliminary tests for making specific filaments for 3D printing with a diameter of 1.75 mm were carried out on a laboratory extruder. The purpose of the experiment was to develop the optimal extrusion temperatures and the speed of drawing the filament to make filaments with rigorously constant dimensions, and the variation in diameter had a maximum of 10%.

## 1. Introduction

In recent years, the field of polymer nanocomposites concerning processing, characterization, and applications has attracted steadily growing interest in the scientific and industrial communities. This significant interest is due to the remarkable properties of polymeric nanocomposite materials compared to the current polymers and conventional macro- or micro-composites [1]. The properties of polymeric nanocomposites (much improved over those conventional materials) refer to elasticity [2,3,4,5,6,7], mechanical resistance, thermal resistance [8], low gas permeability [9,10,11,12,13,14], flammability [15,16,17,18,19], and a high degree of degradability [20].

On the other hand, we have seen a special interest in the theoretical foundation and practical applications regarding the preparation methods and properties of these materials [21,22,23,24,25,26,27,28,29,30,31], which represent unique model systems for studying the structure and dynamics of polymers in restricted or limited environments [32,33,34,35,36,37,38].

Some researchers have tried various techniques for obtaining the composite polymer matrix. [39,40,41,42,43,44,45]. Among these techniques, we mention in situ polymerization and melt-mixing. It is difficult to achieve a universal technique for obtaining polymer composites due to the physical and chemical differences between the systems and the various types of equipment available to researchers. Thus, these different techniques will determine obtaining different results [46].

The addition of rigid filler particles increases the modulus of elasticity proportional to the volume percentage of the filler. The effective fracture surface energy is higher in the composite than in the unfilled polymer. The causes are numerous. The dispersed particles make the crack propagation path longer, increase the plastic deformation of the matrix, and part of the energy is absorbed. So the strength of the composite should increase with increasing filler content. But in reality it doesn’t happen like that because the value of c and the interactions between the neighboring voids predominate.

The value of c represents the size of the voids formed when the matrix detaches from the particles of the filler material due to deformation. Naturally, the larger the particle size of the filler material, the larger the voids will be. It can be concluded that using small particle fillers, finely dispersed, is indicated. Another important conclusion is the anticipation of a considerable statistical dispersion of the strength data for the composite samples because a single void that has acquired a critical size can initiate the main crack.

Practice has also demonstrated the existence of a large number of factors that lead to complications, such as the morphology of the matrix in the composite, dispersed particles as additional crosslinks, the particle structure of the dispersed filler material, porosity, the agglomeration of dispersed filler particles, molecular restructuring of the matrix, and the residual stresses in the composite.

The most noticeable effect of the dispersed filler material on the matrix morphology is expected in the case of semi-crystalline polymers, where the filler largely affects the crystallization conditions. In certain cases, the experimentally observed strengthening effect can be explained by variations in the morphology of the matrix.

This problem has been studied most intensively in the case of elastomers, being found in many publications. It has been established experimentally that a dispersed filler material has a reinforcing effect only if its particle sizes are very small. Obviously, the filler particles are adsorbed by the macromolecules and act as additional points of the macromolecular network. This is realized through a reinforcement of the material. However, it is not possible to explain this effect only by additional crosslinks.

Most researchers believe that the structured particles of the filler interfere with the development of cracks during the deformation of the material.

A secondary side effect of the production process of filled systems is the high void content. Since the presence of voids is due to the initiation of cracks during deformation, it follows that they reduce the strength of the composite. The void content is high in many composites due to poor wetting of the filler particles by the polymer. The presence or release of water on the surface of the particles also favors the formation of voids.

Agglomeration of dispersed filler particles is reflected in the decrease in the mechanical strength of the composite due to the increase in the particle size of the filler material and the low strength of the agglomerates themselves.

In general, processing compositions with a high degree of filling with high viscosity requires high shearing forces and high temperatures. These two factors initiate degradation processes in the matrix, varying the molecular mass distribution of the matrix during the formation process. These effects must be considered when studying the properties of thermoplastic materials with and without filler.

On average, the coefficients of thermal expansion of polymers and mineral fillers differ by a factor of ten. This fact is responsible for the residual stresses that remain in the composite after hardening. Concerning dispersed fillers, the stresses are compressive. Under a tensile force applied, the compressive stresses should manifest an additional resistance to the detachment of the polymer matrix from the filler material, thus improving the strength of the composite. But, in real materials, the structural inhomogeneities give a complex tensional state, which means that at certain points, shear and tensile stresses are present. They can facilitate the propagation of the crack and cause the reduction of the material’s strength.

Thermoplastic Polyurethanes (TPU), due to their high abrasion resistance, strength, chemical resistance, and good fireproof properties, are widely used as insulation for low-voltage electrical cables and for making various profiles by extrusion. [47].

TPUs are linear copolymers with alternating soft and hard segments. The hard segment is composed of diisocyanate (diol or diamine) molecules, while the soft segment consists of a long linear diol chain.

Phase separation occurs in TPU due to the thermodynamic incompatibility of hard and soft segments. The segments aggregate into micro-domains and result in a structure consisting of hard glassy or semi-crystalline and soft rubbery domains, which are below and above the glass transition temperatures (Tg) at room temperature, respectively. The hard domains act as physical crosslinks and impart elastomeric properties to the soft phase. Due to the absence of chemical crosslinking, TPUs can be processed by melting and by solution [48].

Barium titanate (BaTiO_3_) is widely used to manufacture embedded film capacitors. It has relatively low thermal conductivity, very high dielectric constant, and high density, making it unattractive for the preparation of high thermal conductivity dielectric composites [49].

Huang X et al. measured the thermal conductivity of the ethylene–vinyl acetate elastomer with BaTiO_3_ particles (100 nm). They presented a 300% increase in thermal conductivity for an addition of 50% BaTiO_3_ compared to the TPU polymer matrix.

Much research has been carried out to obtain composite materials to improve the properties of TPUs. In one of these studies, TPU/kenaf-type composites were prepared, and the composites showed better mechanical properties than natural TPU [50].

In another study, a new elastomeric composite material based on polystyrene butadiene-styrene (SBS), ester-type polyurethane, and melt-blended polyurethane materials was made. This material presented thermal, dynamic, and mechanical resistance to a large amount of TPU [51].

Other research highlighted the obtaining of new TPU/nano clay composite materials that were characterized after accelerated aging, finding that the TPU/nano clay composite has better mechanical properties than the natural TPU polymer [52].

Recent studies described obtaining flexible transparent electromagnetic interference shielding films with silver mesh fabricated using electric-field-driven microscale 3D printing [53].

Black phosphorus and MXene nanosheets were combined by hydrogen bonding and π- π stacking under ultrasound. Then the as-prepared BP-MXene nanohybrids were coated via the in situ polymerization of dopamine to improve the fire safety and mechanical properties of TPU [54]. Also, a new material 4D printing-encapsulated polycaprolactone–thermoplastic polyurethane with high shape memory performance was obtained by combining the microscopic concept of shape-memory polymers and multimaterial printing of a thermoplastic elastomer with fused deposition modeling without extra operations, such as synthesis and blending [55].

Other researchers obtained antibacterial masterbatches by doping nano-Si_3_N_4_ ceramic materials into PP with a twin-screw extruder. They transferred those masterbatches as raw material into antibacterial meltblown nonwovens during the meltblown process, which were further finished with superhydrophobicity on one side. The as-prepared nonwovens have applications in air filtration, including respirators and masks with a high-efficiency filtration performance and good antibacterial properties [56].

In this paper, composite materials were obtained from TPU/PP/BaTiO_3_ powder and characterized by different methods. The PP + TPU filament variant with 35% BaTiO3 turned out to be the easiest deposition process, with no clogging of the nozzle, no problems with multilayer deposition, and no 3D printing defects. Very good precision structures were obtained experimentally by adjusting the deposition parameters. Precision mesh structures were also made at an angle of 90 compared to the initially planned one of 45 and at deposition densities higher than 50%.

## 2. Materials and Methods

To obtain composite polymers, the following raw materials were used: polypropylene TIPPLEN H 318, thermoplastic polyurethane Estane 58,887 TPU, and BaTiO_3_ powder maximum 2 microns from Sigma Aldrich.

**Figure 1:**
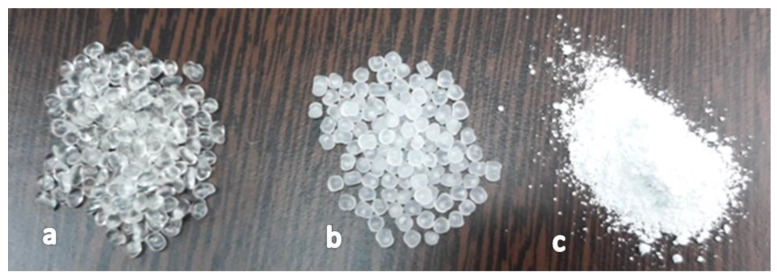
Raw materials used to obtain composite materials: (**a**) TPU beads; (**b**) PP beads; (**c**) BaTiO_3_ powder.

The experimental models were obtained on the KETSE laboratory extruder. In Figure 1, the raw materials used to obtain composite materials of the type TPU/PP/BaTiO_3_ powder are presented.

To obtain good homogenization, the polymers (TPU and PP) and the BaTiO_3_ powder were homogenized by mixing for one hour in a cylindrical mixer with a 1.3 L capacity TURBULA T2F type with a rubber ring holding device, and the rotation speed was 40 rpm. In this way, we sought to obtain a uniform distribution of the components of the mixtures throughout the structure without using specific additives or adhesives for compatibility. Processing conditions involved a rotation speed (in counter-rotation) of the extruder of 95 rpm and a feed speed from the feed hopper of 450 rpm. The main characteristics of the machine are a screw diameter of 28 mm, L/D ratio of 18.6 mm, calculated injection capacity of 58.5 cm^3^, maximum material pressure of 2200 bar, and real injection capacity min 500 mm.

The obtained experimental models have the following codes:

M1—120 g powder of BaTiO_3_ 51 g granules of PP and 829 g granules of TPU (12%)

M2—160 g powder of BaTiO_3_ 49 g granules of PP and 791 g granules of TPU (16%)

M3—200 g powder of BaTiO_3_ 47 g granules of PP and 753 g granules of TPU (20%)

M4—240 g powder of BaTiO_3_ 45 g granules of PP and 715 g granules of TPU (24%)

M5—280 g powder of BaTiO_3_ 43 g granules of PP and 677 g granules of TPU (28%)

M6—320 g powder of BaTiO_3_ 41 g granules of PP and 639 g granules of TPU (32%)

The hydrostatic density was determined by a Mettler Toledo Analytical Balance with these characteristics: maximum capacity 220 g; precision: 0.1 mg; linearity ±0.2 mg; internal calibration; density kit for solids and liquids; the RS 232 interface.

The working temperature was 23.9 °C. The density was determined as the average value between 3 measurements performed on 3 different samples with the exclusion of values outside the range and a confidence level of 95%.

The thermogravimetric analysis and dynamic differential calorimetry (TG/DSC) were carried out with the help of the Simultaneous thermal analyzer TG-DSC type STA 449 F3 (Jupiter, NETZSCH, Germany). This equipment works in the temperature range of −150 °C–1550 °C in an inert, oxidizing, reducing, static, or dynamic working atmosphere. The device is provided with a vacuum system that provides a maximum of 10-2 mbar. (Operating Instructions-Simultaneous TG-DTA/DSC Apparatus (STA 449F3), Jupiter, Selb, October 2008, NETZSCH Gerätebau GmbH).

The conditions of the TG/DSC measurements performed on solid samples of composite polymer materials (10–15 mg) were as follows: temperature range: 25–700 °C, heating speed: 10 K/min, working atmosphere: nitrogen + oxygen, reference substance: alumina.

Before introducing the sample to be analyzed into the device, it is weighed on a digital balance type Precisa XT 220 A (Switzerland), with digital display, precision class 0.1 mg.

Polymer swelling capacity depends on the amount of liquid that the material can absorb when immersed in the liquid. To determine the swelling capacity (swelling) in water and solvent (acetone) for the studied composite materials, the procedure was as follows [57]:

- It weighed approx. 0.06 g of composite material and placed in plastic ampoules with tight caps (tubes for micro-centrifuges with a diameter of 10 mm and a length of 40 mm);
- Two sets of samples were made, one to determine the degree of swelling in water and the other set to determine the degree of swelling in solvent (acetone);
- The ampoules with composite material, thus made, were filled with deionized water and respectively with solvent (acetone) and then were maintained for 72 h at a temperature of 22 °C (atmospheric) and a humidity of 41%.

The following formula was used to determine the degree of swelling:

(1) $$Q=\frac{X_{2}-X_{1}}{X_{1}}*100$$

where:

*Q*—degree of swelling;
*X*_2,3,4_—the mass of the inflated/swollen polymer (after each 72-h cycle);
*X*_1_—dry polymer mass.

## 3. Results and Discussion

From the interpretation of the obtained results, it is found that the melting temperatures vary in the range of 163–166 °C. The temperatures at the beginning of the first oxidation process vary between 190–213 °C.

In Figure 2, Figure 3, Figure 4, Figure 5, Figure 6, Figure 7, Figure 8, Figure 9 and Figure 10, the results of the TG/DSC variation curves as a function of temperature (25–700 °C) are presented for all the studied materials.

**Figure 3:**
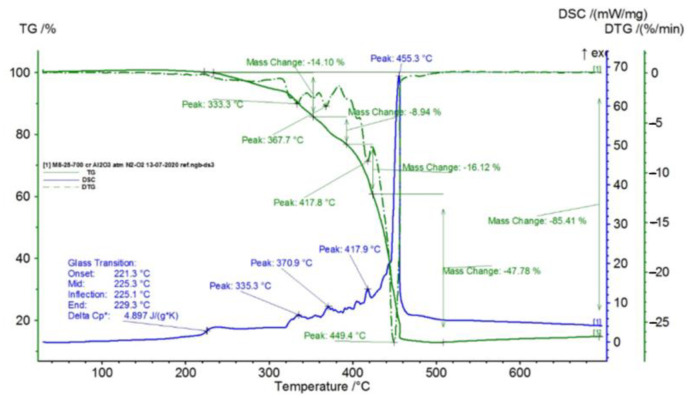
TG/DSC curves registered for TPU.

In Figure 3, the TG/DSC variation curves for the TPU (Thermoplastic Polyurethane) material studied in the temperature range of 25–700 °C are presented. This material presents two transformation processes:

- Glass transition (phase transition of the second order), when the state of rubber changes to the glassy-solid state, with the starting temperature of the transformation at 241.8 °C and variation of ΔCp of 4017 J/g K, a process that occurs due to the presence of amorphous areas in the sample;
- Four chemical oxidation processes with maximum temperatures on the DSC curve at 334.1 °C, 375.9 °C, 408.5 °C, and 523.7 °C, respectively, with corresponding oxidation points on the DTG variation curve, at minimum temperatures of 328.5 °C, 366.4 °C, 409.8 °C, and 528.5 °C, respectively, and with partial mass losses of 32.49%, 23.25%, 19.02%, and 24.70%, respectively, the resulting total mass loss being 99.54%.

**Figure 6:**
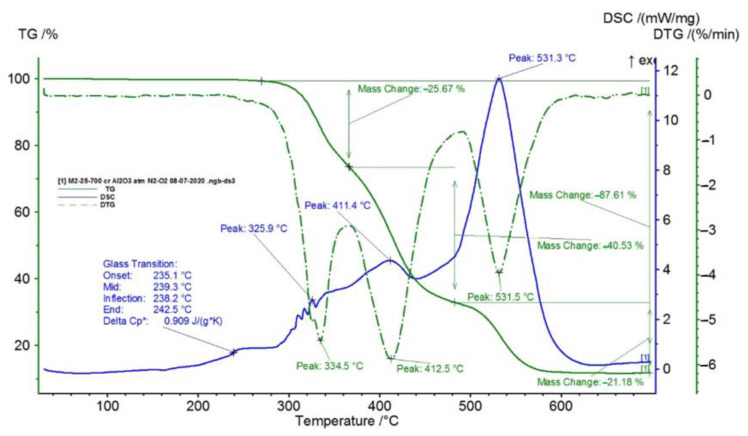
TG/DSC variation for sample M2.

BaTiO_3_ is an inorganic compound with a high melting temperature (1625 °C), which does not present phase transformations in the studied temperature range (25–700 °C), as seen in Figure 6.

In Table 1, the values resulting from the transformation processes (melting, glass transition, oxidation) from the analysis of the TG/DSC variation curves, shown in Figure 2, Figure 3, Figure 4, Figure 5, Figure 6, Figure 7, Figure 8, Figure 9 and Figure 10, are presented.

Composite materials M1–M6 are materials based on BaTiO_3_ powder with the addition of PP (Polypropylene) granules and TPU granules in a constant ratio (approx. 6.2%). In these materials, the concentration of BaTiO_3_ increased from 12% (M1) to 32% (M6), while the addition of thermoplastic polyurethane (TPU) decreased from 88% (M1) to 68% (M6).

**Figure 7:**
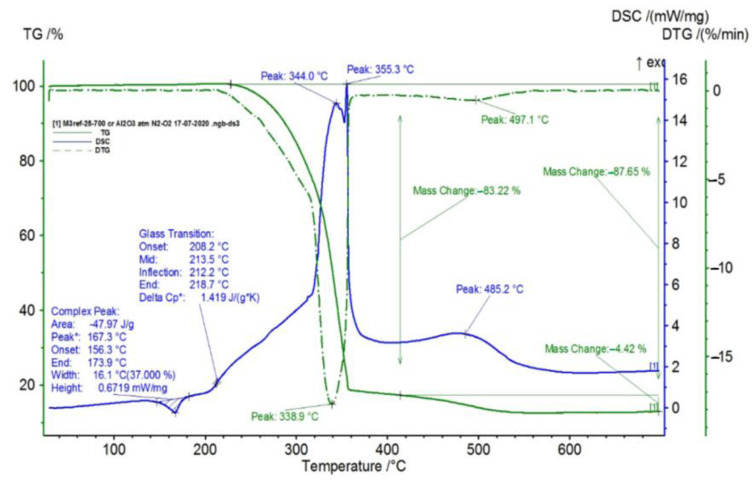
TG/DSC variation for sample M3.

**Figure 8:**
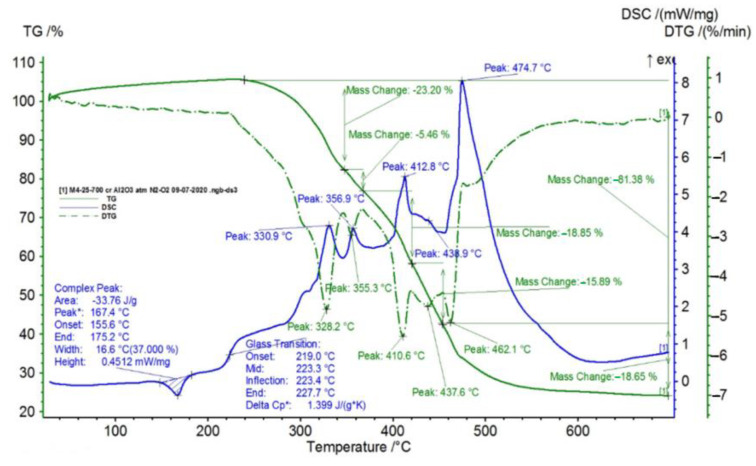
TG/DSC variation for sample M4.

The composite materials M1 and M2 present a thermal analysis behavior similar to that of the TPU material, showing both a glass transition process starting around 230 °C and chemical oxidation processes, with the difference that the second process of oxidation that appears in the TPU material at the maximum DSC temperature of 375.9 °C is no longer present in the composite materials M1 and M2 (Figure 7 and Figure 8).

For composite materials M3–M6, in addition to glass transition processes and chemical oxidation processes, there is also a first-order phase transformation process—melting, due to the polypropylene (PP) melting process. The maximum melting temperature is around 164–167 °C.

Composite material M4 shows the most oxidation chemical processes (5 processes) among all composite materials M1–M6, suffering a total mass loss of 81.38%, which means that this material contains more intermediate products.

Following the TG/DSC analyses performed on materials M1–M6, we can conclude:

- M1 and M2 present a thermal analysis behavior somewhat similar to that of the TPU material, showing both a glass transition process starting around 230 °C and chemical oxidation processes, with the difference that the second process of oxidation that occurs in the TPU material at the maximum DSC temperature of 375.9 °C is no longer present in the composite materials M1 and M2;
- M3–M6, in addition to the glass transition and chemical oxidation processes, there is a first-order phase transformation process—melting using the polypropylene (PP) melting process. The maximum melting temperature is around 164–167 °C;
- M4 presents the most oxidation chemical processes (5 processes) among all composite materials M1–M6, suffering a total mass loss of 81.38%, which means that this material contains more intermediate products.

**Figure 11:**
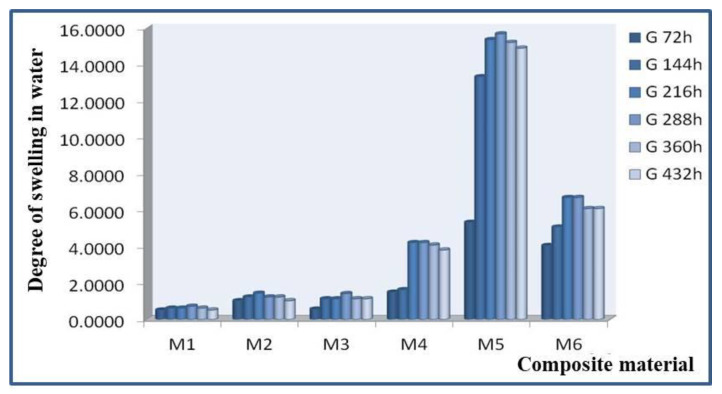
Water swelling of materials M1–M6.

The results of the swelling tests in water for materials M1–M6 are presented in Table 2 and Figure 11.

Experimental data (according to table no. 2) lead to the classification of the composite materials studied from the point of view of the increase in the degree of swelling in water:

- After 72 h in water the degree of swelling varies as follows: M5 > M6 > M4 > M2 > M1 > M3; it can be stated that after immersion in water, the most resistant composite material to the action of water is M3 which shows the lowest degree of swelling;
- After 144 h in water the degree of swelling varies as follows: M5 > M6 > M3 > M4 > M2 > M1; it can be noted that after immersion in water, the most resistant composite material to the action of water is M1 which shows the lowest degree of swelling;
- After 216 h in water the degree of swelling varies as follows: M5 > M6 > M4 > M3 > M2 > M1; it can be stated that after immersion in water, the composite material most resistant to the action of water is again M1 which shows the lowest degree of swelling;
- After 288 h in water the degree of swelling varies as follows: M5 > M6 > M4 > M3 > M2 > M1; it can be stated that after immersion in water, the composite material most resistant to the action of water is again M1 which shows the lowest degree of swelling even the immersion time was longer. However, a beginning of sample saturation is detected by the fact that the degree of swelling increases very little;
- After 360 h in water the degree of swelling varies as follows: M5 > M6 > M4 > M2 > M3 > M1; it can be stated that after immersion in water, the composite material most resistant to the action of water is again M1 which shows the lowest degree of swelling;
- After 432 h in water the degree of swelling stabilizes. It can be noted that after 432 h of immersion in water a saturation of the composite materials occurs.

It is found that starting from 360 h of immersion in distilled water the samples no longer absorb water. Then at 432 h their saturation is observed, and at this moment, the test stops.

**Figure 12:**
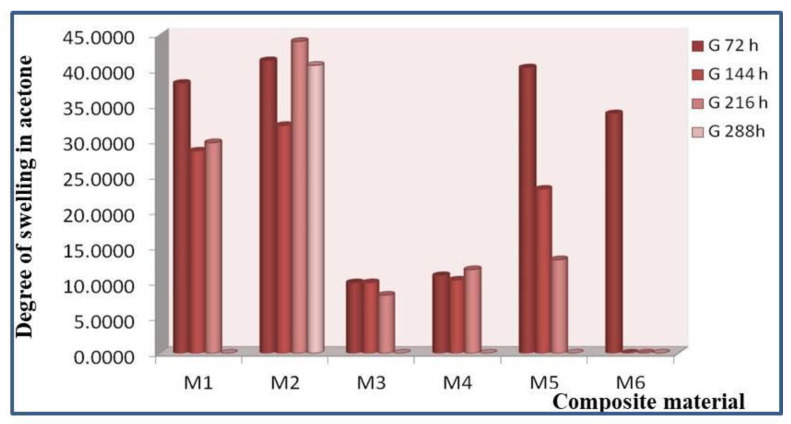
Swelling in solvent (acetone) for materials M1–M6.

The results of swelling tests in acetone for materials M1–M6 are presented in Table 3 and Figure 12.

The degree of swelling was determined by measuring the variation in the mass of the sample. The measurement cycle was 72 h.

Swelling times in acetone were 72, 144, 216, and 288 h.

From the experimental results (Table 3), the following classification of the studied composite materials can be made according to the swelling degree increase in solvent (acetone):

- After 72 h in acetone the degree of swelling varies as follows: M2 > M5 > M1 > M6 > M4 > M3. It can be stated that after immersion in acetone, the most resistant composite material to the action of solvent is M3, which shows the lowest degree of swelling;
- After 144 h in acetone the degree of swelling varies as follows: M2 > M1 > M5 > M4 > M3. It can be noted that after 144 h of immersion in solvent, the most resistant composite material to the action of acetone is M3, which shows the lowest degree of swelling. After 144 h of immersion in acetone, sample M6 disintegrates. At the same time, it can be said that degradation of the composite material also begins, which can be explained by the breaking of some bonds in the composite material;
- After 216 h in acetone the degree of swelling varies as follows: M2 > M1 > M5 > M4 > M3. It can be stated that after 216 h of immersion in solvent, the most resistant composite material to the action of acetone is again M3, which shows the lowest degree of swelling;
- After 288 h, the samples disintegrate and can no longer be tested.

**Figure 13:**
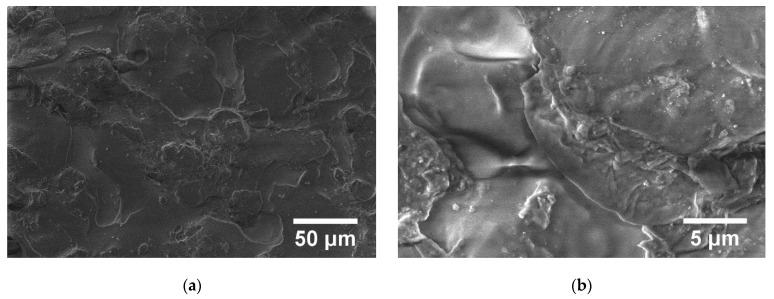
SEM images for PP at magnifications 1.000× (**a**) and 10.000× (**b**).

The structure of the polymer can be seen in SEM pictures. In the case of PP, a melting process can be seen (Figure 13).

**Figure 14:**
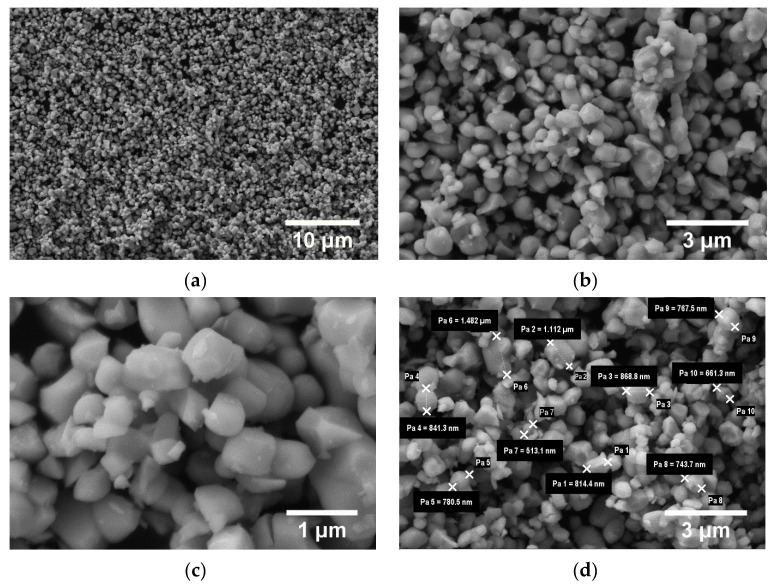

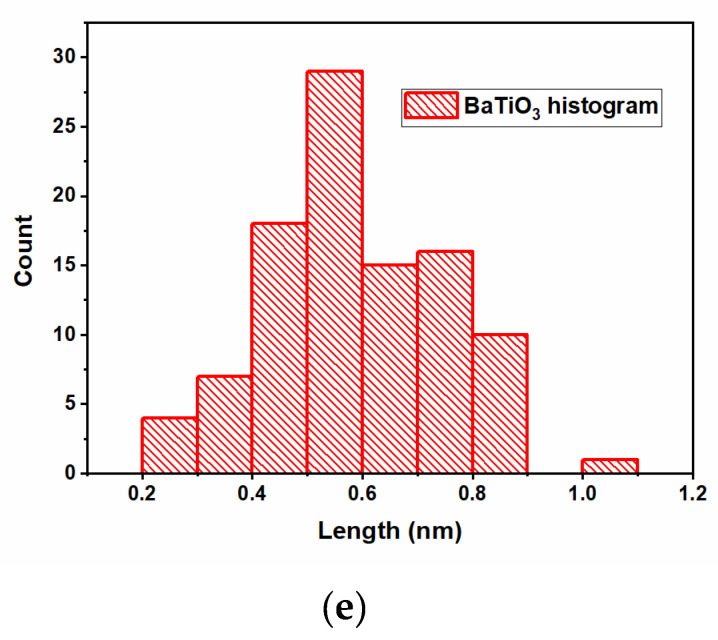
SEM images for BaTiO_3_ powder at magnifications 5.000× (**a**), 20.000× (**b**), 50.000× (**c**); particle sizes (**d**) and histogram of BaTiO_3_ particles (**e**).

For the BaTiO_3_ powder, the micrographs are shown in Figure 14. A large dispersion of particle sizes is found.

The evidence of the differences in the granulation of the BaTiO_3_ powder particles is highlighted in Figure 14. The results of 10 measurements of powder particles are presented, with the following values: 814,4 nm, 1112 nm, 868.8 nm, 841.3 nm, 780.5 nm, 1482 nm, 513.1 nm, 743.7 nm, 767.5 nm, and 661.3 nm. This nano-powder has an average size of 858.46 nm.

**Figure 15:**
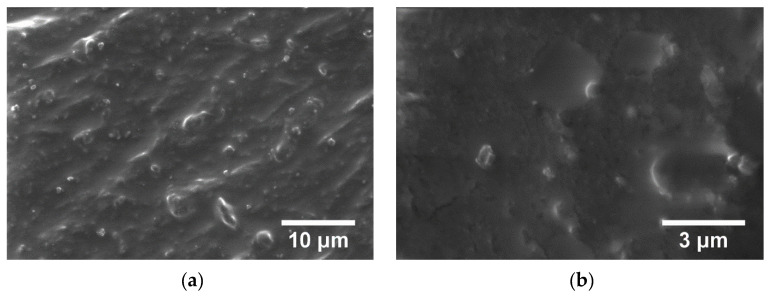

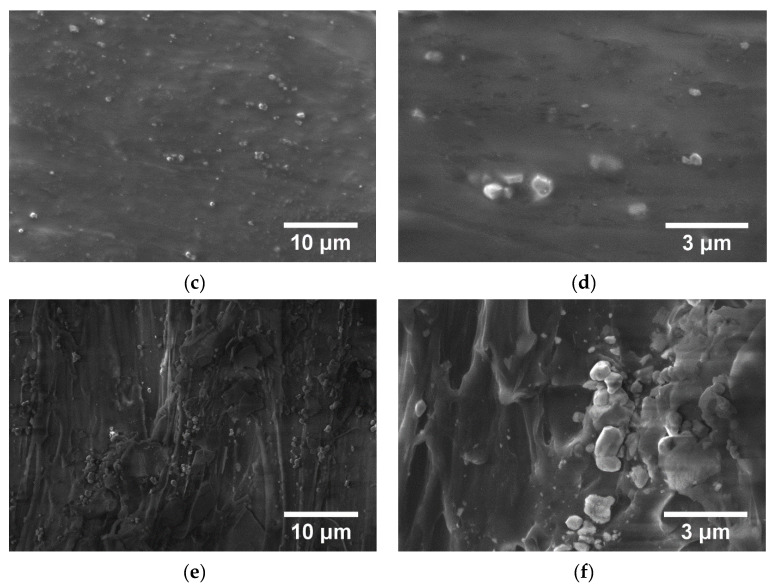
SEM images for composite material M1 at magnifications 5.000× (**a**), 20.000× (**b**), M2 at magnifications 5.000× (**c**), 20.000× (**d**), M3 at magnifications 5.000× (**e**), 20.000× (**f**).

**Figure 16:**
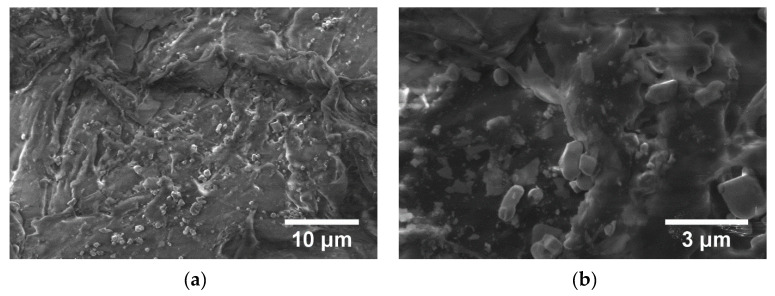

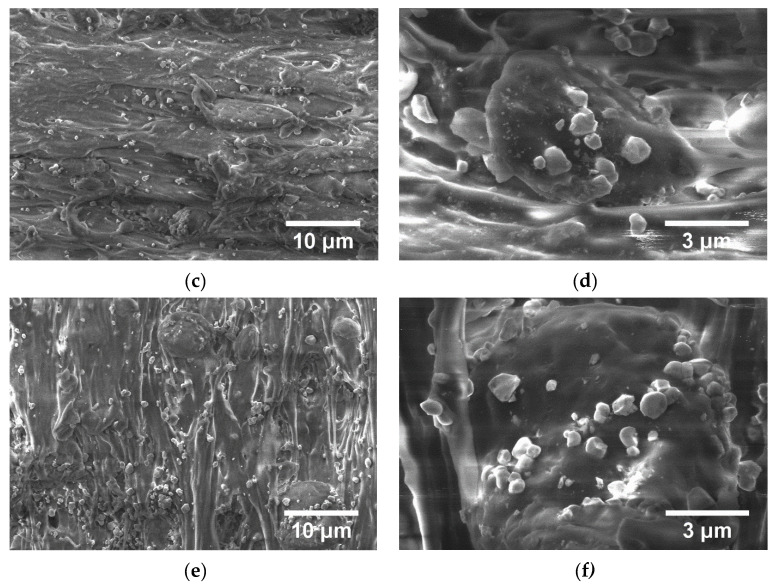
SEM images for composite material M4 at magnifications 5.000× (**a**), 20.000× (**b**), M5 at magnifications 5.000× (**c**), 20.000× (**d**), M6 at magnifications 5.000× (**e**), 20.000× (**f**).

From Figure 15 and Figure 16, the micrographs of TPU/PP/BaTiO_3_ composite materials (M1–M6), powder particles of BaTiO_3_ are observed to be present but not perfectly uniformly dispersed. They form small conglomerates in the polymer structure due to the irregular morphology of the BaTiO_3_ powder that was embedded in the polymer. Analyzing the TPU/PP/BaTiO_3_ type experimental models of composite materials, it is found that M6 is the most homogeneous (respectively, the agglomerations are less).

## 4. Preliminary Processing of Filaments

**Figure 17:**
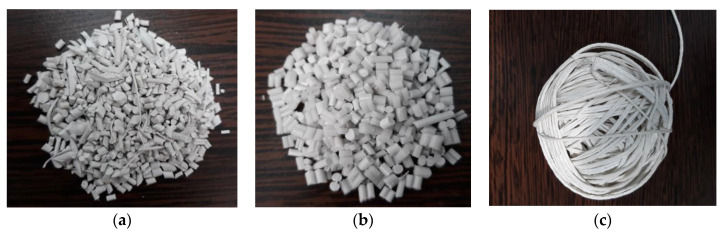
TPU/PP/BaTiO_3_ composite granules (**a**) first pass through the extruder, (**b**) second pass through the extruder, (**c**) the filament.

In Figure 17, the appearance of the granules obtained after one and two passes through the extruder is presented. The filament from the TPU/PP/BaTiO_3_ composite material resulting from the extrusion operation is very elastic, close to the elasticity of rubber, which made the granulation operation difficult and discontinuous (Figure 17c), but the filament itself provided good features to be used for 3D printing applications. The composition of the material (with up to 35% BaTiO_3_) makes it a good candidate for achieving 3D-printed piezoelectric flexible materials for energy harvesting, and the electrical properties are now under evaluation.

**Figure 18:**
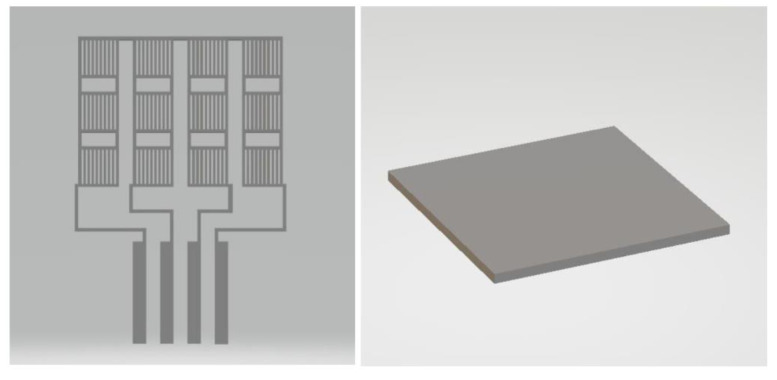
Principle of structural models.

The preliminary tests for making specific filaments for 3D printing with a diameter of 1.75 mm were carried out on a laboratory extruder. The purpose of the experiment was to develop the optimal extrusion temperatures and the speed of drawing the filament to make filaments with rigorously constant dimensions, and the variation in diameter should be a maximum of 10%. The principle of structural models is presented in Figure 18.

The images with the data and the optimal products obtained are reproduced below. For all the recipes tested, the optimal temperatures were around 190 °C with a firing speed of 15 cm/min.

5 types of filaments were made, with the following characteristics:

- 2 work options (injection and extrusion injection)/PP + TPU with 15% BaTiO_3;_
- 2 work options (injection and extrusion injection)/PP + TPU with 25% BaTiO_3;_
- 1 working variant (extrusion) PP + TPU with 35% BaTiO_3._

The preliminary 3D printing tests with the realization of structural models involved using a laboratory 3D thermal printer.

**Figure 19:**
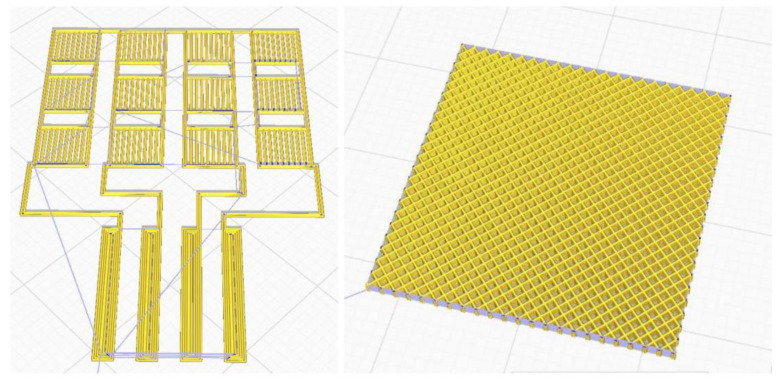
CAD structural models.

The work stages included the following:

- Creation of CAD models on specialized software that are then transferred to the laboratory 3D thermal printer. 2 structural models were designed, one 45 × 30 × 0.5 mm grid type and one 25 × 25 × 1 mm mesh type (Figure 19).

**Figure 20:**
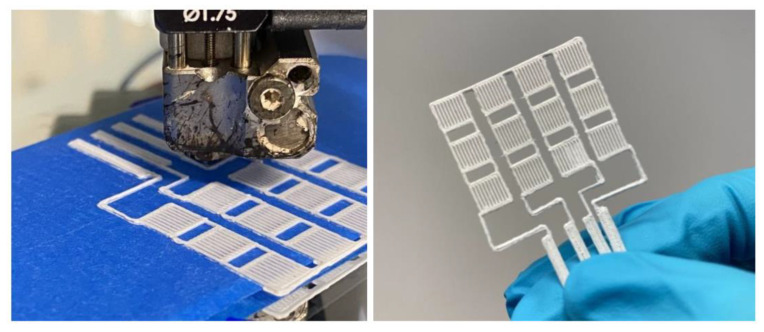

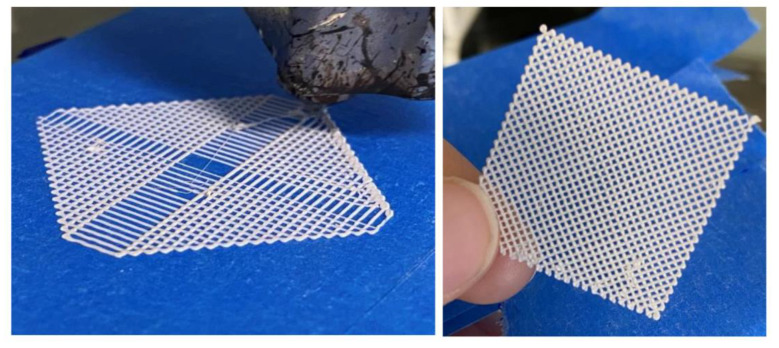
Setting the parameters of the laboratory 3D thermal printer.

Preliminary testing of the 3D printing parameters listed in Table 4, with a comparison between the experimental filaments and the commercial ScotchBlue™ Original Painter’s Tape-type PLA filaments, and with compatibility testing for successive depositions of the two filaments, since the base deposition support can be made with the commercial one (Figure 20).

PP + TPU filament variant with 35% BaTiO_3_ turned out to be the easiest deposition process, with no clogging of the nozzle, no problems with multilayer deposition, and no 3D printing defects.

**Figure 21:**
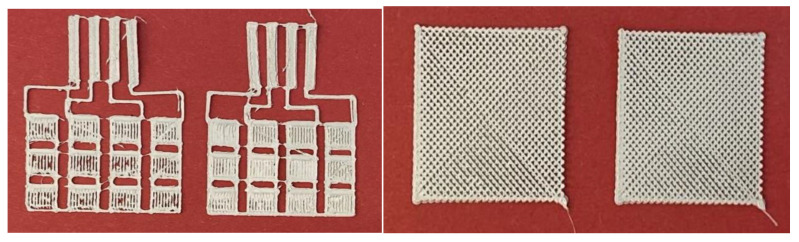
Structures with two deposition densities.

In the end, very good precision structures were obtained experimentally by adjusting the deposition parameters. Even precision mesh structures were made at an angle of 90 compared to the initially planned one of 45 and at deposition densities higher than 50% (Figure 21).

The filament from the TPU/PP/BaTiO_3_ composite material resulting from the extrusion operation is very elastic, close to the elasticity of rubber, a good feature in 3D printing applications. Considering the composite receipt, the material can be a good candidate for achieving 3D-printed piezoelectric flexible materials for energy harvesting.

## 5. Conclusions

In this paper, obtaining and characterizing composite materials from raw materials of the type TPU/PP/BaTiO_3_ powder was presented. The main observations are as follows:

- M1 and M2 present a thermal analysis behavior somewhat similar to that of the TPU material, showing both a glass transition process starting around 230 °C and chemical oxidation processes, with the difference that the second process of oxidation that occurs in the TPU material at the maximum DSC temperature of 375.9 °C is no longer present in the composite materials M1 and M2;
- M3–M6; in addition to the glass transition processes and chemical oxidation processes, there is also a first-order phase transformation process—melting due to the polypropylene (PP) melting process. The maximum melting temperature is around 164–167 °C;
- M4 presents the most oxidation chemical processes (5 processes) among all composite materials, M1–M6, suffering a total mass loss of 81.38%, meaning that this material contains more intermediate products;
- In the case of water inflation, using the M1 composite material is recommended, which has the lowest degree of swelling in water;
- in the case of acetone inflation, the composite materials begin to degrade (dissolve) after immersion for 144 h. Composite M6 disaggregated after immersion for 144 h in acetone;
- Analyzing the TPU/PP/BaTiO3 type experimental models of composite materials, it is found that M6 is the most homogeneous (respectively, the agglomerations are less).

The filament from the TPU/PP/BaTiO3 composite material resulting from the extrusion operation is very elastic, close to the elasticity of rubber, a good feature in 3D printing applications. Taking into account the composite receipt, the material can be a good candidate for achieving 3D-printed piezoelectric flexible materials for energy harvesting.

## Figures and Tables

**Figure 2:**
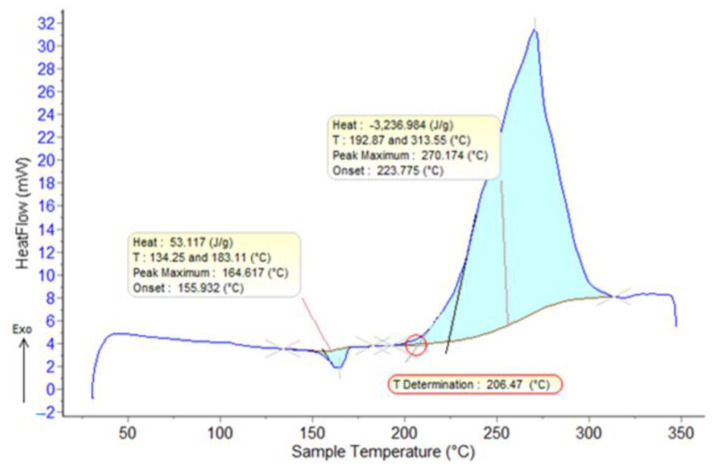
DSC curves (air, 10 °C/min) for PP.

**Figure 4:**
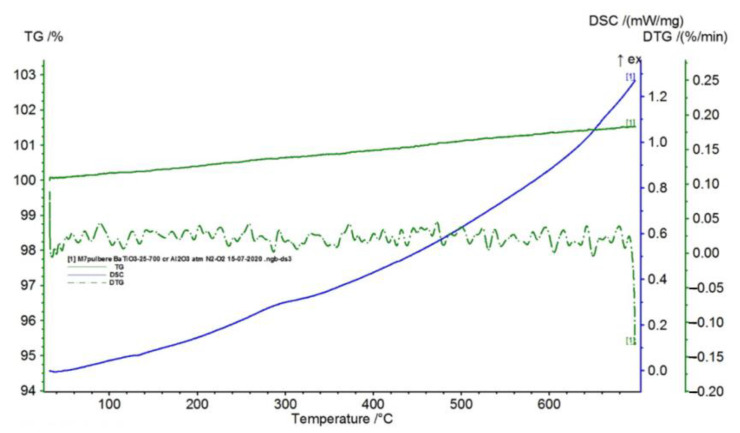
TG/DSC curves registered for BaTiO_3_.

**Figure 5:**
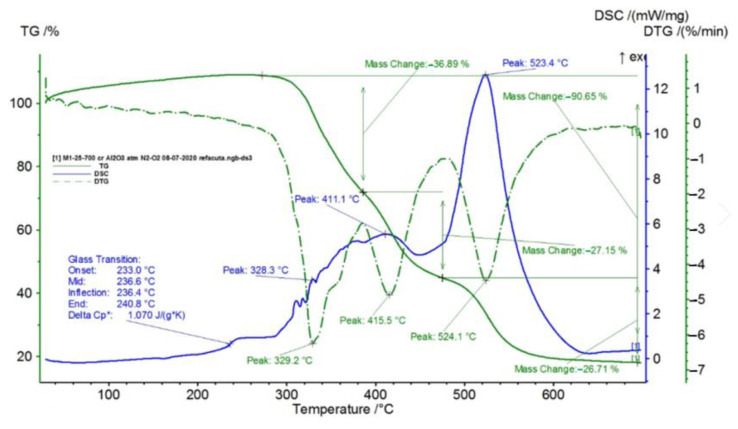
TG/DSC variation for sample M1.

**Figure 9:**
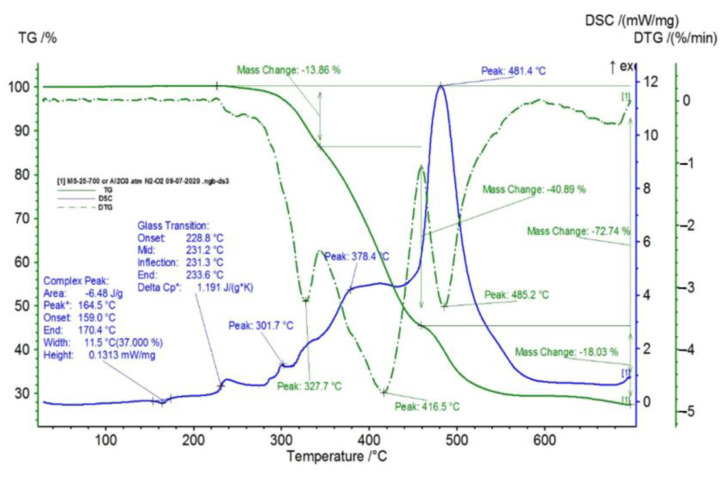
TG/DSC variation for sample M5.

**Figure 10:**
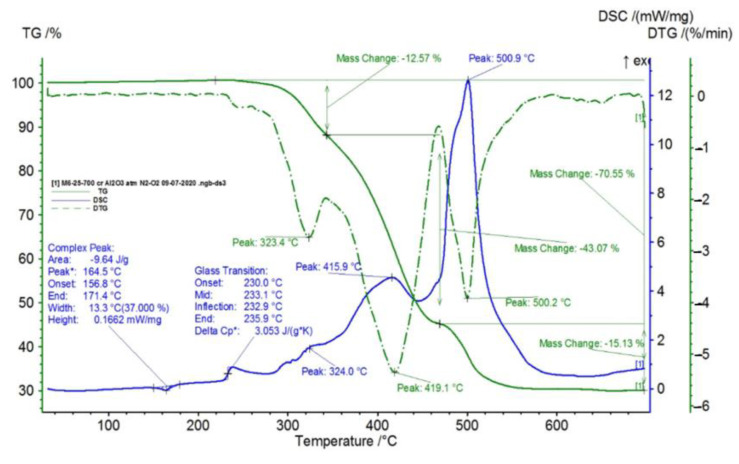
TG/DSC variation for sample M6.

**Table 1:** The hydrostatic density.

Sample	M (mass)	Density			Medium Value	Standard Deviation
		Ethanol Temperature 21 °C				
		1	2	3		
M1	0.121	1.192	1.197	1.217	1.202	0.013
M2	0.131	1.215	1.224	1.210	1.216	0.007
M3	0.141	1.406	1.451	1.424	1.427	0.023
M4	0.211	1.440	1.447	1.429	1.439	0.009
M5	0.189	1.354	1.374	1.374	1.367	0.012
M6	0.191	1.381	1.386	1.374	1.380	0.006

**Table 2:** Experimental results for water swelling of materials M1–M6.

Code	mo	m1	G 72 h	m2	G 144 h	m3	G 216 h	m4	G 288 h	m5	G 360 h	m6	G 432 h
M1	0.0968	0.0973	0.51	0.0974	0.62	0.0974	0.62	0.0975	0.72	0.0974	0.62	0.0973	0.52
M2	0.0487	0.0492	1.02	0.0493	1.23	0.0494	1.44	0.0493	1.23	0.0493	1.23	0.0492	1.03
M3	0.0353	0.0355	0.56	0.0357	1.13	0.0357	1.13	0.0358	1.42	0.0357	1.13	0.0357	1.13
M4	0.0735	0.0746	1.50	0.0747	1.63	0.0766	4.22	0.0766	4.22	0.0765	4.08	0.0763	3.81
M5	0.0638	0.0672	5.33	0.0723	13.32	0.0736	15.36	0.0738	15.67	0.0735	15.20	0.0733	14.89
M6	0.0493	0.0513	4.05	0.0518	5.07	0.0526	6.69	0.0526	6.69	0.0523	6.09	0.0523	6.09

**Table 3:** The swelling tests in acetone for materials M1–M6.

Code	mo	m1	Δm1	G 72 h	m2	Δm2	G 144 h	m3	Δm3	G 216 h	m4	Δm4	G 288 h
M1	0.0764	0.1055	0.0291	38.0890	0.0982	0.0218	28.53	0.0991	0.0227	29.71	solub		0.00
M2	0.0918	0.1297	0.0379	41.2854	0.1213	0.0295	32.14	0.1322	0.0404	44.01	0.1291	0.0373	40.63
M3	0.0171	0.0188	0.0017	9.9415	0.0188	0.0017	9.94	0.0185	0.0014	8.19	solub		0.00
M4	0.0475	0.0527	0.0052	10.9474	0.0524	0.0049	10.32	0.0531	0.0056	11.79	solub		0.00
M5	0.0432	0.0606	0.0174	40.2778	0.0532	0.0100	23.15	0.0489	0.0057	13.19	solub		0.00
M6	0.0728	0.0974	0.0246	33.7912	solub			solub			solub		0.00

**Table 4:** Setting the parameters of the laboratory 3D thermal printer.

Parametru	Grid	Mesh
Layer height	0.2 mm	0.2 mm
Angle of deposition	90	45
Deposit density	100%	50%
Print speed	15 mm/s	30 mm/s
Nozzle diameter	0.4 mm	0.4 mm
Base temperature	60 °C	60 °C
Extrusion temperature	190 °C	190 °C
turns	2 bucle	2 bucle
MULTIPLIER	1.2	1.2

## Data Availability

Not applicable.
